# Isothiocyanates: An Overview of Their Antimicrobial Activity against Human Infections

**DOI:** 10.3390/molecules23030624

**Published:** 2018-03-09

**Authors:** Letizia Romeo, Renato Iori, Patrick Rollin, Placido Bramanti, Emanuela Mazzon

**Affiliations:** 1 IRCCS Centro Neurolesi “Bonino-Pulejo”, Via Provinciale Palermo, Contrada Casazza, 98124 Messina, Italy; letizia.romeo@hotmail.it (L.R.); bramanti.dino@gmail.com (P.B.); 2 Consiglio per la Ricerca in Agricoltura e L’analisi Dell’economia Agraria, Centro di Ricerca Agricoltura e Ambiente (CREA-AA), Via di Corticella 133, 40128 Bologna, Italy; renato.iori48@gmail.com; 3 Institute of Organic and Analytical Chemistry (ICOA), Université d’Orléans et the French National Center for Scientific Research (CNRS), UMR 7311, BP 6759, F-45067 Orléans, France; patrick.rollin@univ-orleans.fr

**Keywords:** isothiocyanates (ITCs), allyl isothiocyanate (AITC), benzyl isothiocyanate (BITC), phenyl isothiocyanate (PITC), phenyl ethyl isothiocyanate (PEITC), sulforaphane (SFN), moringin (MGN), microbial infection, antibiotics

## Abstract

The use of plant-derived products as antimicrobial agents has been investigated in depth. Isothiocyanates (ITCs) are bioactive products resulting from enzymatic hydrolysis of glucosinolates (GLs), the most abundant secondary metabolites in the botanical order Brassicales. Although the antimicrobial activity of ITCs against foodborne and plant pathogens has been well documented, little is known about their antimicrobial properties against human pathogens. This review collects studies that focus on this topic. Particular focus will be put on ITCs’ antimicrobial properties and their mechanism of action against human pathogens for which the current therapeutic solutions are deficient and therefore of prime importance for public health. Our purpose was the evaluation of the potential use of ITCs to replace or support the common antibiotics. Even though ITCs appear to be effective against the most important human pathogens, including bacteria with resistant phenotypes, the majority of the studies did not show comparable results and thus it is very difficult to compare the antimicrobial activity of the different ITCs. For this reason, a standard method should be used and further studies are needed.

## 1. Introduction

Since ancient times, many plants have shown marked beneficial/therapeutic properties for human health [1]. This is mostly due to their content of secondary metabolites, which show strong biological activity, particularly against cancer, cardiovascular and neurodegenerative diseases [2,3,4,5]. It is known that plant metabolites act as naturally-produced antimicrobial agents, inducing self-defense mechanisms to prevent the development of plant infections and to ensure food preservation [6,7,8,9,10,11]. The antimicrobial activity of isothiocyanates (ITCs) against plant and foodborne pathogens has been particularly well documented, together with ITCs’ beneficial properties for human health [8,12,13]. In the plant kingdom, ITCs are produced via enzymatic hydrolysis of glucosinolates (GLs), a class of sulfur-containing secondary metabolites occurring exclusively in the botanical order Brassicales, by the enzyme myrosinase [14,15]. In the intact plant myrosinase is stored separately from GLs. When plant tissue damage occurs by alteration or disruption of tissues caused by chopping or chewing, the myrosinase comes into contact with GLs and effects their hydrolysis and consequently the production of ITCs [12]. It is known that ITCs exert their beneficial properties on human health after the regular consumption in the diet of the edible parts of *Brassica* vegetables rich in GLs.

Although the antimicrobial ability of ITCs to control plant and foodborne pathogen proliferation has been thoroughly investigated [8,12,13], only recently has the action of these compounds to counteract human infections been assessed [12]. Although several studies have described the in vitro antimicrobial activity against bacterial pathogens, little is known about in vivo antimicrobial effects of ITCs. The majority of the investigations refer to the antimicrobial activity of sulforaphane (SFN) against *H. pylori*. Concering this topic, Haristoy et al. used a model of mice implanted with human gastric xenografts, and assessed the antimicrobial activity of SFN after *H. pylori* infection. The results indicated that SFN showed the ability to eradicate acute and recurrent *H. pylori* infections [16]. Moreover, Yanaka et al. used a mouse high salt diet model to evaluate the effect of SFN on gastric atrophy induced by a diet rich in salt after *H. pylori* infection. Results showed that SFN exerted direct antimicrobial activity against *H. pylori* colonization and also induced anti-inflammatory effects protecting the host from gastric mucosal atrophy [17]. 

The remarkable natural properties of ITCs explain the interest in studying these compounds as potential antimicrobial agents against human infections. This statement takes on a greater significance nowadays where the development of new bacteria antibiotic resistance phenotypes is a pressing problem and the pipeline for the development of new antibacterial drugs is now virtually empty.

## 2. Isothiocyanates in Nature

**Scheme 1:**
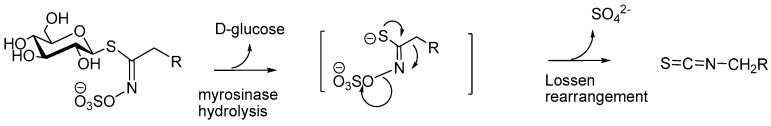
Myrosinase-assisted hydrolytic cleavage of glucosinolates to produce isothiocyanates.

Due to their markedly electrophilic chemical function, ITCs usually show a poor stability in most biological media: e.g., sulforaphane (4-methylsulfinylbutyl ITC) undergoes degradation in many aqueous solvent mixtures [18]. In Nature, ITCs mostly appear in a masked form known as glucosinolates (GLs). Acting generally as bio-precursors of ITCs, GLs are important thiosaccharidic metabolites which occur in all 17 plant families of the order Brassicales, particularly in the predominant family, the Brassicaceae, but also in Capparaceae, Moringaceae, Resedaceae and Tropaeolaceae species [19]. All known GLs—ca. 130 characterized molecules according to Agerbirk [20]—display a remarkable structural homogeneity invariably based on a β-d-glucopyranosyl unit and an O-sulfated anomeric (*Z*)-thiohydroximate function connected to a side chain which constitution, depending on plant species, is the sole structural variant. In Nature, GLs usually co-exist with the enzyme myrosinase (EC 3.2.3.147), the only known glucohydrolase able to break the anomeric C-S bond of GLs [21]. This coexistence, commonly known as the “glucosinolate-myrosinase” system, has been broadly investigated [22,23]. The myrosinase-catalyzed hydrolysis of GLs releases d-glucose and produces aglycones, which can, by expulsion of a sulfate ion, undergo a fast Lossen-type rearrangement to finally deliver the corresponding ITCs (Scheme 1): 

GLs can be divided into several classes according to the chemical structure of their aglycon: aliphatic GLs, like glucocapparin (methyl GL) or sinigrin (2-propenyl GL), thiofunctionalized GLs, like glucoerucin (4-methylsulfanylbutyl GL) or glucoraphanin (4-methylsulfinylbutyl GL), indole-type GLs like glucobrassicin (3-indolylmethyl GL, arylaliphatic GLs, including glucotropaeolin (benzyl GL), gluconasturtiin (2-phenylethyl GL) and glucomoringin (4-(α-l-rhamno-pyranosyloxy)benzyl GL) [19,20]. A similar classification can be given for the corresponding ITCs: aliphatic, like methyl or allyl ITC (methyl or allyl mustard oils), thiofunctionalized, like erucin (4-methylsulfanylbutyl ITC) or sulforaphane (4-methylsulfinylbutyl ITC), arylaliphatic like benzyl and 2-phenylethyl ITC. Last but not least, the more structurally complex compound moringin (4-(α-l-rhamnopyranosyloxy)benzyl ITC), obtained from glucomoringin should also be placed into the arylaliphatic class (Table 1). 

The beneficial impact of GL-containing vegetables on human health has received much attention and ITCs produced by enzymatic hydrolysis of GLs, which are present in edible parts, are regarded as most likely to maintain human health through their regular consumption in diets [24]. A recent study has provided evidence that enhancing the GRA content in soups made with novel broccoli varieties results in increased levels of SFN in human plasma [25]. Following absorption, SFN is metabolized through the mercapturic acid pathway and excreted in urine, predominantly as an *N*-acetylcysteine conjugate [25]. Recently, a great deal of attention has been paid to the Moringaceae family, due to their bioactive ingredients ubiquitously available in every edible part of the plants—leaves, seeds, bark, roots, exudates, flowers, and pods [26]. A number of reports on MGN, the ITC derived from glucomoringin isolated from *Moringa oleifera*, highlight various biological activities such as anticancer [27], anti-inflammatory [28], antioxidant [29] and also antibacterial, antifungal and antiviral activities [30,31,32]. Moreover, ITCs produced from an extract of *Cleome chrysantha* showed good antimicrobial activity against Gram-negative bacteria, moderate activity against Gram-positive bacteria, and a good inhibitory activity against the growth of *Saccharomyces cerevisiae* [33]. *Capparis ovata* and *Capparis spinosa* extracts were similarly investigated for their antibacterial activity [34].

## 3. ITCs Metabolism 

The metabolism of GLs in the human body occurs by their absorption through the gastrointestinal mucosa after *Brassica* vegetable diet ingestion. However, the majority part of the ingested GLs is metabolized in the gut lumen. Specifically, when un-processed *Brassica* vegetables are consumed, the active myrosinase enzyme (present in these plants) hydrolyzes the GLs and produce different metabolites, including ITCs, but also nitriles, oxazolidine-2-thiones, and indole-3-carbinols. Intact GLs and GL-derived breakdown products such as ITCs are absorbed in the proximal part of the gastrointestinal tract (stomach and small intestine). GLs derived from cooked *Brassica* vegetables (containing inactive plant myrosinase) transit to the colon (due to their intact hydrophilic nature, consisting of thioglucose and sulfate group) where they are hydrolyzed into the GL-derived breakdown products by the intestinal microbiota. Therefore, the cecum and colon are the sites where GL-breakdown products, including ITCs, are adsorbed after the intestinal microbiota processing [35]. Moreover, rapid ITC absorption occurs in blood (the peak is observed 3 h after ingestion) and in different tissues [36,37]. The highest amount of ITCs are found in the intestinal mucosa, in the liver, in the kidneys, and in the bladder, followed by the lungs and the spleen. The brain and the heart contain very low concentrations of ITCs [35]. Concerning ITCs’ excretion, the absorbed ITCs arrive at the liver where they are conjugated with glutathione and finally excreted in the urine as mercapturic acid. The amount of mercapturic acid excreted is correlated with the amount of ITCs consumed [35]. Specifically, the bioavailability of ITCs and consequently the ITCs’ excretion depend on the mode of GL hydrolysis and on the content of Gls ingested with the diet. Indeed, it has been observed that the consumption of raw *Brassica* vegetables improves ITCs’ bioavailability compared to cooked vegetables. The hydrolysis of GLs in raw vegetables is due to plant myrosinase. This process determines an amount of mercapturic acid excretion of 17–88% of the dose and type of GLs ingested (chemical structure of GLs and type of ingested plant such as mustard, cabbage, watercress, broccoli, etc.) [35,38]. On the other hand the amount of mercapturic acid excreted after the consumption of cooked *Brassica* vegetables accounts for only 20% of the ingested GLs [35,39]. 

Interestingly, another way to improve bioavailability of ITCs was reported by Sivapalan et al., where the consuption of soup made with novel broccoli varieties rich in GLs enhanced the delivery of sulforaphane to the systemic circulation [25].

## 4. Isothiocyanates Antimicrobial Properties against Human Pathogens

Since the early 1900s, great interest has been shown in plant antimicrobial properties. Walker reported in 1925 on plants’ resistance to infection, providing a scientific contribution describing the ability of onion bulbs to resist fungal infections [40]. The interest in plant antimicrobial properties is related both to improving the cultivation and conservation of edible plants and to understanding their role in human health. For this reason, many articles focus on plant metabolites with antibacterial properties [41,42]. Only recently, the lack of new antibiotics and subsequent failure to provide resolute therapy against multi-drug-resistant microbes has increased the need of an intense search for innovative solutions. This review focuses on ITCs’ antimicrobial properties against human pathogens, especially against bacteria with multi-drug-resistance phenotypes for which the discovery of a new therapeutic solution is more pressing. The antimicrobial activity of ITCs against the microbes selected in this review is summarized in Table 2. 

### 4.1. Helicobacter Pylori

*Helicobacter pylori (H. pylori)* is a Gram-negative bacterium that colonizes human gastric mucosa, causing gastritis and peptic ulcer disease, a condition that may be associated with the development of gastric cancer. *H. pylori* infection is difficult to eradicate, and therapeutic failure is due to the onset of resistant strains which persist in the gastrointestinal tract in a non-growing tolerant form or as an intracellular pathogen [69,70,71,72].

In 2002 Fahey et al. investigated the antibacterial properties of sulforaphane (SFN) against *H. pylori* [43]. Obtained from broccoli seeds, SFN was used to determine the minimal inhibitory concentration (MIC) and minimal bactericidal concentration (MBC) against three reference strains (*H. pylori* 26695, J99 and ATCC 43504) and 45 clinical isolates of *H. pylori* from patients with gastritis and gastric or duodenal ulcers. Regarding the 45 clinical isolates, six were resistant to both antibiotics (CLA^R^Met^R^ strains). The results indicated that SFN showed an interesting high inhibitory activity (median MIC = 2 μg/mL) against bacterial strains resistant to two antibiotics, clarithromycin and metronidazole. In addition SNF showed similar antibacterial activity against sensitive strains (median MIC = 2 μg/mL) compared to resistant strains, suggesting that SNF’s antimicrobial activity was not dependent on bacteria phenotype [43]. Furthermore, SFN was able to kill the intracellular forms of *H. pylori*—responsible for *H. pylori* persistent infections—after only 24 h of treatment [43]. This result could be of great clinical value because one of the reasons for the clinical treatment failure is the onset of resistant strains which persist as intracellular pathogens, as indicated before [43].

SFN’s antibacterial effects against *H. pylori* were also confirmed by in vivo experiments [16]. Haristoy et al. [16] demonstrated that natural SFN induced complete eradication of *H. pylori* from human gastric xenografts implanted in nude mice [16]. The authors report that eight of 11 xenografts treated with SFN, showed completely eradicated *H. pylori* infections.

The mechanism of action of SFN against *H. pylori* was recently investigated [73]. It is well known that *H. pylori* gastric infection is ensured by its ability to survive in the acidic stomach environment by producing a wide range of ureases [74] which convert urea into ammonia which neutralizes the acidic environment of the stomach [75]. These evidences led Fahey et al. to investigate a possible correlation between SFN’s bactericidal activity and *H. pylori* urease inactivation [73]. However, the experimental data did not suggest any functional relationship between those properties. Further investigations will have to be conducted to clarify the molecular mechanism involved in SFN’s antimicrobial activity against *H. pylori* [73].

### 4.2. Clostridium Difficile and Clostridium Perfringens

The genus *Clostridium* is composed of several different Gram-positive, endospore-forming, anaerobic species, including two enteric pathogens, *Clostridium perfringens* and *Clostridium difficile*, that can threaten human health [76]. Ingestion of contaminated food, dietary factors and the imbalance of intestinal microflora induced by antibiotic therapy [77,78] can cause *Clostridium* infections [79,80]. The prevalence and severity of *C. difficile* infections have boosted research efforts to detect new virulence factors and to develop new treatments and prevention regimens.

In order to identify phytochemical compounds that preserve the natural intestinal microflora and ensure pathogen strain growth inhibition, Kim and Lee [47] evaluated the antimicrobial activity of phenethyl isothiocyanate (PEITC) purified from *Sinapis alba* seed oil against pathogenic *Clostridium* species [47]. The results demonstrated that 2 mg/disc of PEITC inhibited *C. difficile* ATCC 9689 and *C. perfringens* ATCC 13124, with an inhibition zone >30 mm. The authors did not compare these results with those obtained using conventional antibiotics so the potential clinical efficacy of PEITC against infections of both *Clostridium* strains cannot be indicated. Furthermore, hypothetical inhibitions towards the commensal bacteria (*Bifidobacterium bifidum*, *B. breve, B. longum, Lactobacillus acidophilus* and *L. casei*) were evaluated and PEITC was shown not to affect the intestinal microflora. In order to understand if the chemical structure of ITCs may influence their antimicrobial activity against *Clostridia* species, the authors [47] compared the results obtained using natural PEITC from *Sinapis alba* seed oil extract with synthetic ITCs containing aromatic hydrocarbons (benzyl and benzoyl groups) and aliphatic hydrocarbons (acetyl-, allyl-, butyl-, ethyl- and methyl groups). Antimicrobial activity of natural PEITC was comparable to synthetic BITC using a similar dose (2 mg/disc). On the contrary, benzoyl ITC showed a decrease in growth-inhibiting activity against *Clostridia*. No growth inhibition properties were also observed using aliphatic ITCs such as AITC. These results suggested that aromatic ITC were favorites due to their ability to cross bacterial membrane structures and they probably could exert their antimicrobial activity better than aliphatic ITCs. Moreover, regarding aromatic ITCs, the decrease of inhibition activity observed for benzoyl ITC—that only differs from benzyl isothiocyanate by the presence of a carbonyl (-C=O) group—suggested that ITCs showed the highest activity against *Clostridia* when only one benzyl group is involved [47].

Regarding the resistant strains of *Clostridia,* Park and Choi [81] assessed the antimicrobial activity of a horseradish root extract (containing 59.9% AITC, 35.8% PEITC and 1.5% 3-butenyl ITC) against *C. perfringens* KCTC 3269 resistant strain compared to chlorhexidine digluconate used as a control [81]. The natural extract (MIC = 6.67 mg/mL and MBC = 16.67 mg/mL) inhibited *C. perfringens* KCTC 3269 growth, but its efficiency was lower than that of the control antibiotic (MIC = 0.13 mg/mL and MBC = 0.21 mg/mL). In addition the authors evaluated the contribution of AITC to the slight antimicrobial property of horseradish root by comparing the antimicrobial activity of the natural extract with synthetic AITC (MIC = 8.33 mg/mL and BMC = 8.33 mg/mL). Natural extract and synthetic AITC showed similar antibacterial activity, suggesting that the AITC is the main horseradish ITC responsible for the slight antibacterial effect, despite the fact its effectiveness is very low. Although the antimicrobial assay and the used strains were not comparable, these results appear to be in agreement with Kim and Lee [47] who indicated the inability of the aliphatic AITC to counteract *Clostridia* species.

### 4.3. Campylobacter Jejuni

*Campylobacter jejuni* is the main cause of human enteric infections in developed and developing countries [82]. Over the years, new undescribed *Campylobacter* species were identified, and the increased number of *Campylobacter* antibiotic-resistant strains [83,84] has promoted research activities aimed at finding new therapeutic approaches.

In order to analyze the antimicrobial activity of ITCs against *C. jejuni* strains, Dufour et al. [53] tested the antimicrobial property of the following commercially available ITCs: AITC, BITC, ethyl ITC, and 3-methylsulfanylpropyl ITC. Each of those ITCs was evaluated against 24 *C. jejuni* strains including the reference strain NCTC 11168 and clinical isolates from various origins (chicken feces, human infections—blood or feces—and contaminated processed meats) some of these with different antibiotic-resistant phenotypes [53]. BITC was the most effective ITC against sensitive and resistant bacteria included in the study. Indeed only 1.25 μg/mL of BITC was capable of counteracting the growth of the most sensitive *C. jejuni* and only 5 μg/mL of BITC was sufficient to inhibit the growth of nine more resistant strains. AITC was also active against all the bacteria, but its efficacy was less than that of BITC (200 μg/mL of AITC were required to exert a similar effect to BITC against resistant *C. jejuni* strains). No positive results were obtained by testing the other ITCs included in the study. These findings were indicative of a marked antimicrobial activity of BITC against *C. jejuni* and a higher antimicrobial potential compared to AITC [53].

In addition, in order to clarify the BITC antibacterial mechanism of action against *C. jejuni* the transcriptomic profiles of *C. jejuni* NCTC11168 treated with BITC were analyzed using a microarray approach [54]. Results indicated that BITC treatment induced an increased expression levels of genes involved in the heat shock response pathways such as *clpB* (ATP-dependent dis-aggregase), *hrcA* (heat-inducible transcriptional regulator), *dnaK* (chaperone), *grpE* (co-chaperone of *dnaK* and nucleotide exchange factor), *groES* (chaperone), *cbpA* (*dnaJ*-like protein) and *Cj0760* (hypothetical protein, metallo-β-lactamase superfamily). Furthermore, BITC determined the upregulation of *trxA* (thioredoxin) gene involved in iron-sulfur cluster homeostasis and *sodB* (superoxide dismutase) gene involved in the oxidative stress response. Additionally, BITC stimulated the upregulation of genes involved in energy metabolism such as *gapA* (glyceraldehyde-3-phosphate dehydrogenase A) gene, *fba* (fructose-bisphosphate aldolase) gene, *frdC* (fumarate reductase, cytochrome b-556 subunit) gene and *frdB* (fumarate reductase, iron-sulfur subunit) gene. Those results suggested that the antimicrobial action of BITC is exerted through triggering pathways inducing to heat shock and oxidative stress response, protein aggregation and to the dysfunction of energy metabolism leading to bacterial death [54]. 

### 4.4. Salmonella Enterica

*Salmonella* are Gram-negative, flagellar and optionally anaerobic bacilli belonging to the Enterobacteriaceae family. All medically important *Salmonella* are included in the *S. enterica* species and are divided into typhoidal *Salmonella* serotypes and non-typhoidal *Salmonella* serotypes [85].

The main studies carried out to evaluate the antimicrobial properties of ITCs against *S. enterica* species were referred to the total extracts or oil and not to the isolated or synthetic ITCs. The following studies evaluated the antimicrobial effect of different extracts from three species of *Moringa* plant [59,65,67] (*Moringa oleifera*, *Moringa peregrina* (Forssk) and *Moringa stenopetala*) and of the oil obtained from *Salvadora persica* [52]. The authors investigated the part of the plant that showed the most effective antibacterial activity and the most useful extraction method to obtain a phyto-compound able to exert the greatest antimicrobial property against sensible and resistant *S. enterica* strains. The antimicrobial properties of aqueous, methanol and chloroform extracts of *Moringa oleifera* leaves, bark and roots against *S. enterica* were recently investigated [65]. The results indicated that the bark chloroform extract showed the highest antimicrobial activity against *S. enterica* reference strain compared to the other extracts (IZ > 20 mm) [65]. Moreover, *n*-hexane extracts of *Moringa oleifera* and *Moringa stenopetala* seed showed antimicrobial activity against *S. enterica* [67]. 

Regarding to the *S. enterica* strains with resistant phenotype, Saleh et al. [59] investigated the antimicrobial potential of *Moringa peregrina* (Forssk) seed extracts against 35 *S. enterica* clinical isolates with resistant phenotype. Specifically four of them were characterized for MDR phenotypes resulting insensible to fluoroquinolones that are currently used as the last defense therapy against non-typhoid *S. enterica* infection.The study was performed comparing the antimicrobial properties of the extracts of *M. peregrina* seeds obtained using *n*-hexane or *n*-butanol or water. Results indicated that the aqueous seed extract of *M. peregrina* showed an excellent inhibitory effect (MIC ranging from 109.37 to 437.5 mg/mL based on the different clones tested) against resistant isolates including strains with MDR phenotype. Moreover it was the most effective extract compared to *n*-hexane and *n*-butanol extracts [59]. 

In order to evaluate the bacterial ultrastructural changes induced by the aqueous extract of *M. peregrina* seed the authors compared treated and non-treated bacteria (used as negative control) by scanning and transmission electron microscopy (STEM). Electron micrographs showed that treatment with the aqueous extract induced a modification of the bacterial surface with formation of holes in some parts of the cell wall. This outcome advanced with a time-dependent manner with a progressive structural disorganization of the cytoplasm and the appearance of wavy membranes. The cell wall was finally disrupted and leakage of cellular components was observed, with subsequent cell death [59]. Although the antimicrobial effect was shown observing electron micrographs the mechanism behind the mode of action is still unknown.

Finally Sofrata et al., suggested that BITC—the main component of *Salvadora persica* root oil—displayed antimicrobial effects against *S. enterica.* The authors mainly characterized the essential oil obtained from *Salvadora persica* root indicating that the oil contained 73.8% of BITC and 26.2% of benzyl cyanide and then tested its antimicrobial effect against *S. enterica* ATCC14028 ser. *Typhimurium* [52]. Results indicated that oil rich in BITC exhibited high antibacterial activity in a dose-dependent manner against *S. enterica* ATCC14028 ser. *Typhimurium.* Specifically 0.02% of oil extract was sufficient to significantly reduce *S. enterica* CFU counts and 0.1% of the oil extract was able to completely abrogate *S. enterica* growth. In addition, oil-exposed bacteria were analyzed by transmission electron microscopy (TEM) to observe the bacterial ultrastructural changes induced by *S. persica* root oil treatment. The experiment was performed comparing the effect of the 0.1% of the oil (containing 2.8 µmole BITC) and similar amount of synthetic BITC (2.8 µmole) to simulate its contribution into the oil. Electron micrographs revealed dramatic oil-induced-effects on the bacterial membrane, which exhibited small protrusions after only two minutes of incubation. The size of these protrusions increased in a time dependent manner, leading to loss of bacterial membrane integrity. In addition, synthetic BITC (2.8 µmole) induced similar protrusions on the bacterial membrane compared to those observed by *S. persica* root oil treatment. Altogether, these results suggested that BITC was the most active compound against *S. enterica* in *Salvadora persica* root essential oil exerting its effect on the bacterial membrane, although further studies could be done to understand the mechanism of the antibacterial action [52].

### 4.5. Escherichia coli

*Escherichia coli* is the main facultative anaerobic bacterium that lives in the human intestine. Although considered a harmless commensal microorganism, it can be responsible for severe intestinal and extraintestinal infections. Several strains of adapted *E. coli* have been identified and characterized. New clones have shown the ability to acquire virulence factors that make *E. coli* able to colonize different human districts and cause a wide range of diseases [86]. The most investigated intestinal pathogenic strain of *E. coli* is enterohemorrhagic *E. coli* (EHEC) [87]. The outcome of EHEC infections depends on the production of Shiga toxins (Stx). The strains of *E. coli* that produce Stx (STEC), such as *E. coli* O157: H7, have been studied extensively [88]. ITCs are currently considered as potential therapeutic agents against EHEC infections [45].

Recently Lu et al. assessed the antimicrobial property of an extract of *Wasabia japonica* (Japanese horseradish) containing 59 μg/mL of AITC against *E. coli* O157:H7 (B0241 strain), comparing it with its synthetic analogue. The authors reported that 59 μg/mL of natural AITC was sufficient to inhibit *E. coli* growth up to 12 h of incubation and a similar result was obtained using the synthetic analog, suggesting that AITC is the main ITC responsible for the antimicrobial activity of *Wasabia japonica* [55].

Lin et al. evaluated the antibacterial efficacy of synthetic AITC compared to polymyxin B against *E. coli* O157: H7 ATCC 43895 strain [56]. Results indicated that synthetic AITC (1.000 μg/mL) showed less efficacy than polymyxin B (10 μg/mL). Indeed a 100-fold greater amount of AITC was required to exert a similar result as the conventional antibiotic. Moreover, AITC induced its antimicrobial effect on bacterial membranes, leading to leakage of cellular substances and to bacterial death similar to that observed for polymyxin B [56]. Although this experiment suggested that AITC induced its antimicrobial effect on the bacterial membrane, the mechanism of action of AITC against *E. coli* O157:H7 is still unknown. For this reason Luciano and Holley assessed the inhibitory effect of synthetic AITC toward two enzymes having a key role in the bacterial metabolism: thioredoxin reductase, implicated in ribonucleotide synthesis; and acetate kinase, related to energy metabolism [57]. The conclusions reached by the authors indicated that AITC inhibits the level of activity of thioredoxin in a dose-dependent manner. It was hypothesized that AITC may interact with the sulfhydryl groups in the catalytic domain of thioredoxin, inducing inferior enzyme activity that could influence bacterial DNA synthesis. Although less effective, AITC was also able to significantly inhibit acetate kinase activity through the sulfhydryl groups of the enzyme, influencing bacterial energy metabolism [57]. 

AITC is not the only ITC that has been shown to exert antimicrobial effects against *E. coli* strains. Indeed Pal et al. reported that ethanolic *Moringa* leaf extract showed antimicrobial activity against *E. coli* [60]. Subsequently, Rahman et al. tested the effectiveness of powdered *Moringa* leaf juice and leaf extracts (water or ethanol) against *E. coli* isolated from patients [61]. The results indicated that only the powdered leaf juice had bactericidal efficacy (IZ = 40.45 ± 0.37) when compared to the tetracycline control (IZ = 19.03 ± 0.44). The water or ethanol extracts showed a lower efficacy compared to powdered leaf juice but they are comparable—fresh leaf aqueous extract IZ = 16.8 ± 0.12; ethanol extract IZ = 21 ± 0.16—to the conventional antibiotic.

Moreover, Borges et al., reported that both synthetic AITC and PEITC were able to affect *Escherichia coli* CECT 434 with similar effects [49]. The authors also indicated that AITC and PEITC were capable of affecting the bacterial cell membrane integrity in a dose-dependent manner. In addition AITC and PEITC increased the hydrophilic character of the membrane changing the physico-chemical characteristics of the bacterial membrane [49].

Recently, Nowicki et al. showed that BITC was more effective than AITC in the inhibition of EHEC *E. coli* growth [45]. The study was performed comparing the antibacterial activity of synthetic SFN, AITC, BITC, phenyl ITC (PITC) and isopropyl ITC (IPRITC) against different clinical and laboratory EHEC *E. coli* strains, including WT (*E. coli* MG1655) and Shiga toxin-producing *E. coli* (O157:H7 *E. coli)*. Results indicated that BITC displayed the highest inhibitory growth ability and the highest bactericidal activity against wild type and Stx-producing *E.coli* strains compared to the other assessed ITCs. Specifically, BITC showed MIC = 0.07 mg/mL and MBC = 0.15 mg/mL against WT EHEC *E. coli* strain compared to AITC showing MIC = 0.4 mg/mL and MBC = 0.79 mg/mL against the same strain. Furthermore, BITC showed similar MIC values against WT *E. coli* as well as for clinical isolates carrying Shiga toxin-converting prophages. All the other ITCs showed similar trends although with less efficacy compared to BITC. The only minor exception was SFN, which was more effective against the 86-24 strain carrying Shiga toxin converting prophages than the other strains, although its activity was in any case inferior to BITC [45]. It is known that the conventional antibiotic treatment against EHEC *E. coli* can induce the production of Shiga toxins from bacteria. For this reason, the authors tested whether AITC, BITC, PITC, SFN and IPRITC could produce similar effects. Results indicated that the ITCs included in the study (AITC, BITC, PITC, SFN and IPRITC) alone did not induce prophage development exerting a protective effect on the Shiga toxin production [45]. This is one of the most important findings because ITCs could be used not only to replace antibiotics but also as a combined treatment with them.

Finally the ITCs’ mechanism of action against *E. coli* Shiga toxin production strains was also investigated [45]. Specially the authors evaluated the effect of ITCs (AITC, BITC, PITC, SFN and IPRITC) on the “alarmone” penta/tetraphosphate (p)ppGpp that was able to influence the activity of RNA polymerase and the development of bacteriophage and Shiga toxin production. Results indicated that all tested ITCs included in the study induced an increase in (p)ppGpp "alarmone" level and indirectly downregulate RNA synthesis affecting prophage development and consequently the Shiga toxin production [45].

### 4.6. Pseudomonas Aeruginosa

*Pseudomonas aeruginosa* are aerobic Gram-negative bacteria that can colonize different environments. *P. aeruginosa* is an opportunistic pathogen that is able to regulate biofilm formation and the release of different virulence factors in response to cell-to-cell signaling and environmental stimuli. Pathogenic and non pathogenic bacteria are able to produce biofilms. This statement plays an important role in the social communication of bacteria and their ability to survive in the natural environment. Although biofilms exert a positive role in a variety of ecosystems they are often associated to the survival of pathogens during antibiotic treatment leading to worse clinical outcomes [89]. Indeed, it is known that *P. aeruginosa* strain biofilm producers show a greater resistance to common antibiotic treatment [90,91,92] and they are often associated to medical device colonization and nosocomial infections [93]. Many bacteria, including *Pseudomonas* strains are equipped with a quorum sensing (QS) system able to control biofilm production and the release of different virulence factor in response to environmental stimuli. Specifically, the QS system detects environmental stimuli and releases signal molecules, defined as “auto-inducers” that contribute to the microbial pathogenicity [94]. At least two major QS systems—*las* and *rhl*—have been identified in *P. aeruginosa*. The *las* QS systems is composed by *lasI*, the autoinducer synthase gene responsible for the synthesis of the correlate (*N*-[3-oxododecanoyl]-l-homoserine lactone autoinducer, and the *lasR* gene that codes for a transcriptional regulator that determines the transcription of QS genes including virulence factors.The *rhl* system similarly consists of the pair RhlI/RhlR, which responds to *N*-butyrylhomoserine lactone (C4-HSL) [94,95]. QS in *P. aeruginosa* regulates not only biofilm production, but also the release of important virulence factor such as pyocyanin that affects the central nervous system, the urolological and the vascular system inducing inflammatory effect [96] and rhamnolipid involved in the *P. aeruginosa* dissemination across human ephitelia [97]. 

Over the years, multi-resistant strains of *P. aeruginosa* biofilm producers have been isolated from patients. The acquisition of new resistance phenotypes underlines the need for new antibiotic therapeutic strategies and anti-biofilm molecular compounds [90,92,93,98,99].

The antimicrobial activity of total extracts and powdered juice from *Moringa oleifera* leaf (fresh and dried) against *P. aeruginosa* were investigated by Rahman et al. [62]. The aim of the study was to determine if fresh or dried leaf from *Moringa oleifera* was able to counteract the growth of *P. aeruginosa* (BMLRU1017) strain and also what was the most effective extraction method that ensure the best growth inhibition. Rahman et al. indicated that *Moringa oleifera* leaf showed antimicrobial activity against *P. aeruginosa* and especially the powder obtained from fresh leaf juice (1175 μg/disc) had a higher inhibitory effect (IZ = 39.60 ± 0.49 mm) compared to tetracycline control (IZ = 18.30 ± 0.12 mm). Regarding the extracts, only fresh leaf (1175 μg/disc) displayed antimicrobial activity, and no inhibition halo was observed using extracts from dried leaf. Moreover ethanol extract from fresh leaf showed strong antibacterial effect (IZ = 21.21 ± 0.05) comparable to the antibiotic. Fresh leaf cold water extract (1175 μg/disc) displayed only a slight antibacterial effect (IZ = 15.00 ± 0.34 mm), while hot water extracts did not display antimicrobial activity [62]. 

Recently the effects of synthetic ITCs against different clinical isolates of *P. aeruginosa* were investigated by Kaiser et al. [48]. The authors tested the antimicrobial properties of synthetic AITC, BITC and PEITC against 105 clinical isolates of *P. aeruginos*a characterized to show from two to six times greater ability to produce biofilms and resistant phenotypes [48]. Tests were also performed using a mixture (38% (*v*/*v*) AITC, 50% BITC and 12% PEITC) indicating as ITCM. The results showed that AITC was the most effective ITCs against the *P. aeuriginosa* clinical isolate, followed by the ITCM mixture, by the BITC and finally by PEITC. Subsequently AITC, BITC, PEITC and the ITCM mixture were used to test their ability to inhibit biofilm production. Results indicated that PEITC (at a concentration of 500 μg/mL) caused a significant reduction in the biofilm development of *P. aeruginosa* (native biofilm formation of 1.02 ± 0.04 versus 0.62 ± 0.05 under the influence of PEITC) and only 100 μg/mL of the ITCM mixture were sufficient to achieve similar result (1.02 ± 0.04 of native biofilm formation versus 0.69 ± 0.06 in treatment with the mixture). BITC and AITC did not show inhibitory effect on biofilm production. To the contrary, AITC was the most effective ITC (concentrations ranging between 200 and 800 μg/mL) against *P. aeruginosa* strains into the mature biofilm followed by BITC (concentrations from 250 to 1000 μg/mL) and finally by ITCM mixture (concentrations from 500 to 1000 μg/mL). The less effective ITC was the PEITC in contrast to what observed for *P. aeruginosa* strains during biofilm development [48]. Moreover it has been observed that ITCM exposure induced the killing of bacteria into the mature biofilm without restriction to biofilm permeation, one of the most critical point for the effectiveness of any antimicrobial agent used against mature bacterial biofilm. These are interesting finding because anti-biofilm compound that show both the ability to prevent biofilm formation and the ability to inhibit metabolic activity within established biofilms are rare. Moreover seems to be no such diffusion barrier for the tested ITC compounds as suggested by the authors. The authors also reported a synergistic effect combining the ITCM mixture with meropenem against biofilms produced by carbapenem-susceptible *P. aeruginosa* clinical isolates. Interestingly meropenem alone was more efficient than ITC mixture to killing planktonic bacteria but only when combined with ITCM mixture it was possible to observe an substantial reduction of carbapenem-susceptible *P. aeruginosa* metabolic activity into the biofilm. In contrast, no synergistic effect of meropenem and ITCM mixture on biofilms produced by carbapenem resistant *P. aeruginosa* strains was observed [48].

The antimicrobial effects of these ITCs on *P. aeruginosa* growth has been recently investigated by Borges et al. [49]. The authors tested the effect of AITC and PEITC against *P. aeruginosa* ATCC 10145 strain. Results indicated that AITC (MIC = 100 μg/mL) and PEITC (MIC = 100 μg/mL) were capable of altering the integrity of the bacterial cell membrane in a dose-dependent manner. In addition the treatment with AITC and PEITC determined an increase in the hydrophilic character of the membrane, thereby changing the physico-chemical characteristics of the bacterial membrane [49]. Although these results indicated that ITCs antibacterial activity against *P. aeruginosa* occurred by alterations of cell membrane, this evidence did not explain the mechanism of action against *P. aerugoinosa* strains. 

An alternative strategy to suppress the growth of *P. aeruginosa* lies in inhibition of the bacterial quorum sensing (QS). Concerning this evidence Ganin et al [44] investigated about the ability of synthetic SFN and ERN to activate or interfere with the *las* and *rhl* QS systems of *P. aeruginosa.* Results indicated that SFN and ERN were shown to inhibit bacterial quorum detection. The *P. aueruginosa* QS system inhibition induced by ITCs was indirectly confirmed evaluatingthe effects of the ITCs on biofilm formation and on the production of pyocyanin—a virulence factor of *P. aeruginosa*—both processes being controlled by the QS system. The authors [44] indicated that both SFN and ERN showed to be very effective to reduce the production of pyocyanin and interestingly showed good imanti-biofilm activity, although SFN displayed a greater efficacy compared to ERN. To explain this result Ganin et al. [44] hypothesized that the differences between SFN and ERN could be attributed to the different hydrophobicities of those ITCs, resulting in different permeability and distribution capacity during the growth of the biofilm. An alternative explanation may be a slower oxidation of ERN into SFN in the biofilm environment.

Furthermore the effect of iberin (IBN) on *P. aeruginosa* QS was also investigated by Jakobsen et al. [58]. The authors have shown that IBN, extracted from horseradish, was able to down-regulate the expression of the RhlI/RhlR QS network in *P. aeruginosa*. IBN was also able to downregulate the QS-controlled virulence factors by activating the genes involved in the production of pyocyanin and genes (rhlA and rhlB) involved in the production of rhamnolipid [58]. The latter contributes to *P. aeruginosa* infection through their surfactant capacities leading to killing polymorphonuclear leukocytes. In particular, the RhlR (PA3477) part of the *P. aeruginosa* QS network appeared to be significant (>5-fold) down-regulated in the bacterial culture treated with IBN (64 μg/mL). RhlI was only moderately (about 4-fold) down-regulated, while LasR (<2-fold downregulated) and LasI (<2-fold upregulated) seemed to be practically unaffected [58].

### 4.7. Staphylococcus Aureus and Methicillin-Resistant S. aureus (MRSA)

*Staphylococci* are Gram-positive bacteria that are commonly found on the skin and hair, as well as in the respiratory tract of many people, without causing infection or disease. *Staphylococcus aureus* (*S. aureus*) however is commonly associated with serious skin and systemic infections, especially in people with cancer, vascular and pulmonary diseases [100,101]. The greater incidence of *Staphylococcus* invasive infections is registered in hospitalized patients, due to the compromised immune system, to the surgery procedures and to the use of medical devices [102,103]. These parameters make *S. aureus* one of the main pathogens that cause nosocomial infections. Over the years, the rise of *S. aureus* phenotypes resistant to antibiotics such as methicillin has been recorded [104].

*Moringa oleifera* antimicrobial activity against *S. aureus* has been investigated in depth [30,62,63,64,66,68]. Rahman et al. [62] assessed the antimicrobial properties of *Moringa oleifera* leaf extract against *S. aureus* (BMLRU1002 strain) indicating that water extracts from fresh leaf displayed similar antibacterial effect (IZ = 12.00 ± 0,12 mm) to the antibiotic tetracycline used as a control (IZ = 12.76 ± 0.02) [62]. This property was also confirmed by Peixoto et al. (IZ = 25.4 mm) [63]. In addition Vieira et al. [66] investigated about the antimicrobial activity of *M. oleifera* seed extracts against *S. aureus.* The authors indicated that aqueous (IZ = 25 mm) and ethanol (IZ = 28 mm) extracts from *Moringa oleifera* seeds showed similar antimicrobial activity against *S. aureus* ATCC 25923 strain, exerting their activity in a dose-dependent manner [66]. Furthermore, seed extracts obtained with dichloromethane allowed Padla et al. [30] to demonstrate that the two structurally-related ITCs isolated from the *M. oleifera* seed extract—4-(α-l-rhamnopyranosyloxy)benzyl ITC (moringin) and its 4′-*O*-acetylated derivative (4′-Ac-moringin)—showed to inhibit *S. aureus* ATTC 6538 growth with inhibition halo similar to those observed for the canonical antibiotic ofloxacin [30]. As demonstrated by Galuppo et al [68] moringin was also able to counteract the growth of *S. aureus* BAA-977 strain (clindamycin-resistant strain) with an inhibition halo of about 25 ± 1 mm. Finally, ethyl acetate extract of *M. oleifera* bark showed the higher antimicrobial activity against *S. aureus* (MTCC-740 strain) compared to methanol and chloroform extracts [64].

These studies were done using different *S. aureus* strains and different doses of the ecxtracts determining a difficult comparison of the results and an incorrect evaluation of the best extract against *S. aureus*. Despite this evidence, altogether these studies suggested that *Moringa oleifera* showed good antimicrobial activity against *S. aureus* and moringin was a promising structurally-related ITC to develop further investigations. 

On another note, the antimicrobial effect of synthetic AITC, BITC, PEITC, SFN, and a mixture of AITC, BITC and PEITC (containing 3 μmol of each ITC)—on *S. aureus* was also investigated [50]. Aires et al., [50] demonstrated that the mixture of ITCs showed the most effective antimicrobial activity against *S. aureus* clinical isolate followed by BITC, PEITC and finally by SFN. No inhibition effect was observed for AITC. In addition, BITC, PEITC, SFN and the ITC mixture displayed a better antimicrobial activity compared to vancomycin (IZ = 18 mm). It is worth noting that the ITC mixture showed inhibitory activity similar to BITC alone, which suggests that BITC is the best inhibiting compound in the ITC mixture and that no synergistic effect arises with AITC and PEITC [50].

Similar results were obtained by Dias, et al. [46]. The authors tested the antimicrobial activity of AITC, BITC, PEITC, SFN, ERN and IBN and their combined effect with the vancomycin antibiotic. Results indicated that synthetic BITC and PEITC induced a total inhibition of the *S. aureus* strain CECT 976 (standard MRSA strain) growth showing the best antimicrobial activity. Regarding the other ITC included in the study, PEITC and ERN showed better antimicrobial activity than IBN which in turn was more effective than SFN. No effect was observed for AITC. In addition all the effective ITC showed a better antimicrobial activity compared to vancomycin [46]. 

Finally, the synergic effect of a combined treatment of each ITCs with vancomycin against *S. aureus* CECT 976 was also evaluated [46]. As expected, both BITC and PEITC retained their strong antibacterial activity indicating that the combined treatment improved the antibacterial effect of vancomycin against *S. aureus* CECT 976. Moreover the association of ERN and vancomycin improved the antimicrobial activity of each one replace similar results to BITC and PEITC. Finally the association of IBN with the antibiotic had no effect [46].

Although AITC has appeared not to have antimicrobial activity against *S. aureus*, Lu, et al. [55] recently showed that AITC-rich *Wasabia japonica* powder was able to induce a significant reduction of *S. aureus* B0031 growth in a dose dependent manner [55] and similar effects were observed by using the synthetic analog AITC (10 μg/mL < MIC < 100 μg/mL), which suggests that AITC is the most active *Wasabia japonica* component to inhibit *S. aureus* B0031 growth.

In addition Borges et al. [49] indicated that AITC and PEITC showed antibacterial activity against *S. aureus* CECT 976 with similar effect (MIC = 100 μg/mL). The author also reported that AITC and PEITC treatment altered the bacterial cell membrane integrity lead to bacterial death [49].

A few years ago, Dias et al. [51] evaluated the antibacterial properties of synthetic AITC, BITC and PEITC on resistant phenotypes (MRSA). The experimental work involved 15 clinical isolates of methicillin-resistant *S. aureus* and one standard strain (*S. aureus* CECT 976). Results indicated that BITC and PEITC showed higher inhibition activity compared to vancomycin and to AITC. More specifically, 10 isolates were more susceptible to PEITC, two isolates were more susceptible to BITC and two were highly susceptible to both BITC and 2-PEITC. Furthermore, AITC and PEITC were essentially bacteriostatic while BITC was bactericidal in 11 MRSA isolates (representing 69% of isolates tested). Based on these results, BITC was more effective than PEITC in suppressing MRSA strains. The authors hypothesized that variations in the chemical structure of ITCs might explain the different grade and type of antibacterial activity, and they postulated that phenyl rings present in BITC and PEITC structure could be a predisposing factor for antibacterial efficacy.

## 5. Conclusions

The review suggests that the most effective ITCs against the human pathogens included in the collected studies, are the SFN, AITC, BITC and *Moringa*. Interestingly, these ITCs exert a broad spectrum of action against Gram positive and Gram negative bacteria, although AITC appears to be more effective against Gram negative bacteria, specially against *E. coli* strains, while BITC and *Moringa* are more active against *S. aureus*, including multi-drug-resistance strains. Finally, SFN showed strong activity against *H. pylori*. Furthermore, the ITCs’ antimicrobial mechanism of action occurs by affecting membrane integrity and enzymes involved into the redox balance and bacteria metabolism up to determine the bacteria death. The majority of the studies carried out, are performed by in vitro experiments, instead little is known about the effects of ITCs in vivo. Concerning this statement further studies could be done to evaluate the potential therapeutic use of the ITCs.

## Figures and Tables

**Table 1:** Most investigated bio-active ITCs and other hydrolysis products from GLs.

Glucosinolate	Acronym	Side Chain	Derived ITC	Acronym
Glucocapparin	GCA	Methyl	Methyl ITC	MITC
Sinigrin	SIN	2-Propenyl	Allyl ITC	AITC
Glucoerucin	GER	4-Methylsulfanylbutyl	Erucin	ERN
Glucoraphanin	GRA	4-Methylsulfinylbutyl	Sulforaphane	SFN
Glucotropaeolin	GTL	Benzyl	Benzyl ITC	BITC
Gluconasturtiin	GST	2-Phenylethyl	Phenethyl ITC	PEITC
Glucomoringin	GMG	4-(α-l-Rhamnopyranosyloxy)benzyl ITC	Moringin	MGN
Glucobrassicin	GBS	Indol-3-ylmethyl	Indole-3-carbinol (*)	I3C

(*) indol-3-ylmethyl ITC undergoes fast hydrolysis to the corresponding alcohol.

**Table 2:** Major ITCs showing antibacterial activity and mechanism of action.

ITCs	ITCs Source	Sensible Bacterial	Mechanism of Action	Reference
**Sulforaphane** **SFN**	broccoli seed extract	*H. pylori*	NA	[43]
**Sulforaphane** **SFN**	Synthetic	*P. aeruginosa*, EHEC *E. coli* strains, *S. aureus*	SFN inhibits bacterial quorum sensing, affects the pyocyanin production and exerts anti-biofilm activity against *P. aeruginosa*. SFN inhibits EHEC *E. coli* Shiga toxin production.	[44,45,46]
**Phenethyl isothiocyanate** **PEITC**	*Sinapis alba* seed	*C. difficile* ATCC 9689 and *C. perfringens* ATCC 13124	PEITC mechanism of action against *Clostridia* species appears to be correlated with aromatic structure.	[47]
	Synthetic	*P. aeruginosa*; *S. aureus*, *E. coli CECT 434*	PEITC affects *E. coli* CECT 434, *P. aeruginosa* ATCC 10145, *S. aureus* CECT 976 cellular membrane integrity. PEITC reduce *P. aeruginosa* biofilm development.	[46,48,49,50,51]
**Benzyl isothiocyanate** **BITC**	*Salvadora persica* root	*S. enterica* serotype *Typhimurium*	Essential oil rich in BITC, induces the loss of bacterial membrane integrity.	[52]
**Benzyl isothiocyanate** **BITC**	Synthetic	*C. difficile*; *C. perfringens*; *C. jejuni*; EHEC *E. coli*; *P. aeruginosa*; *S. aureus*	BITC inhibits EHEC *E. coli* Shiga toxin production. BITC induces the loss of MRSA membrane integrity and potential. BITC influences C.jejuni redox balance and metabolism up to death. BITC mechanism of action against *Clostridia* species appears to be correlated with aromatic structure. BITC reduces the metabolic activity of *P. aeruginosa* into the mature biofilm	[45,46,47,48,50,51,53,54]
**Allyl isothiocyanate** **AITC**	*Wasabia japonica*	*E. coli* O157:H7; *S.aureus*	NA	[55]
**Allyl isothiocyanate** **AITC**	Synthetic	*C. jejuni E. coli* O157:H7; *E. coli* CECT 434 *P. aeruginosa*; *S. aureus*	AITC affectes *E. coli* O157: H7 cell membrane integrity. AITC displays inhibitory action against thioredoxin reductase and acetate kinase of *E. coli* O157:H7 interacting with the sulfhydryl groups of the enzymes. AITC inhibites EHEC *E. coli* Shiga toxin production. AITC affects *E. coli* CECT 434, *P. aeruginosa* ATCC 10145, *S. aureus* CECT 976 cellular membrane integrity.AITC aliphatic structure impares its ability to counteract MRSA growth compared to the other ITCs. AITC reduces the metabolic activity of *P. aeruginosa* into the mature biofilm.	[45,48,49,51,53,55,56,57]
**Erucin** **ERN**	Synthetic	*P. aeruginosa*; *S. aureus*	ERN inhibits *P. aeruginosa* quorum sensing nd affects the production of pyocyanin.	[44,46]
**Iberin** **IBN**	*Armoracia rusticana*	*P. aeruginosa*	IBN inhibits *P.aeruginosa* quorum sensing and affects the production of pyocyanin and rhamnolipid	[58]
**Iberin** **IBN**	Synthetic	*S. aureus*	NA	[46]
	*Moringa peregrina* seed extract	*S. enterica* isolates including MDR strains	Moringa peregrina aqueous seed extract induces the loss of cell wall integrity membrane potential.	[59]
	*Moringa oleifera* leaf extract	*E. coli*, *P. aeruginosa S. aureus*	NA	[60,61,62,63]
	*Moringa oleifera* bark extract	*S. aureus*, *S. enterica*	NA	[64,65]
	*Moringa oleifera* seed extract	*S. aureus*, *S. enetrica*	NA	[66,67]
***Moringin*** **MGN**	*Moringa oleifera* seed extract	*S. aureus*	NA	[30,68]
	*Moringa stenopetala* seed extract	*S. enterica*	NA	[67]
